# From Local Pilots to National Implementation: A Journey Towards Free HPV Vaccination in China

**DOI:** 10.3390/vaccines14060528

**Published:** 2026-06-15

**Authors:** Yinqi He, Yihan Fu, Zhitao Wang, Jing Sun

**Affiliations:** School of Health Policy and Management, Chinese Academy of Medical Sciences & Peking Union Medical College, Beijing 100730, China

**Keywords:** HPV vaccination, National Immunization Program, pilot, implementation, China

## Abstract

China recently became the 155th country to provide free vaccination to all 13-year-old girls with two doses of a domestic bivalent HPV vaccine in October 2025. Such a policy change aligns with the Immunization Agenda 2030, which expects more investment of domestic resources into immunization rather than heavily depending on external donor funding support. This review examines the policy-making evolution process and analyzes how the final decision was made at the national level, using the Multiple Streams Framework. Unlike traditional NIP expansion, which adopts a top-down decision-making strategy, China’s free HPV vaccination policy evolved with a distinct bottom-up strategy originating from local pilots, which is demonstrated to be instrumental for national policy-making. The extensive local pilots of free HPV vaccination have served as a powerful engine that drives a rapid and substantial increase in HPV vaccination rate, played a pivotal role in shaping the market of HPV vaccines, and contributed to achieving the economies of scale, which triggered a substantial price reduction. It also fostered a national consensus on the critical role of HPV vaccination in cervical cancer prevention and control, a principle now enshrined in the core public health knowledge repository across the country. A potential strategy to introduce new vaccines into the NIP could be piloting first and expanding incrementally with the bottom-up strategy, leveraging a comprehensive platform under the framework of the national policy, and then making use of the effect of scale and peer pressure, high level engagement, cross-departmental collaboration, and multiple financing mechanisms.

## 1. Background

Cervical cancer, primarily caused by persistent human papillomavirus (HPV) infections, represents the most significant disease burden among HPV-related cancers [1]. It is one of the few malignancies that can be effectively prevented through vaccination [2]. The World Health Organization (WHO) recommends member states to introduce the HPV vaccine into the National Immunization Program (NIP) for girls aged 9–14, and sets a target that 90% of girls be fully vaccinated by age 15 [3]. Countries around the world have been actively responding to this call. At the time of writing this analysis, the HPV vaccine has been introduced into the NIP of 155 out of 194 (80%) WHO member states [4].

Given its large population, China bears a substantial burden of cervical cancer. Nearly 150,000 new cases of cervical cancer and 50,000 related deaths were reported in China in 2024 [5], accounting for 22% and 16% of the global totals, respectively [6]. Among women aged 15 to 44, cervical cancer ranked third in both incidence and mortality in China [7]. Higher incidence and mortality of cervical cancer were reported in less developed Northwest and Southwest provinces, compared with the developed areas like Beijing and Shanghai [5]. Unlike the declining trends in both the incidence and mortality rates of cervical cancer observed in high-income countries, China has been experiencing a rising incidence rate alongside a younger onset trend [8]. However, China had been lagging behind in introducing the HPV vaccine into the NIP, constrained by the capacity of local production and supply of HPV vaccines, and considerable expenditure for NIP expansion. The HPV vaccination rate for girls aged 9–14 was only 1.9% before 2020 [9]. The cumulative HPV vaccination rate among Chinese women aged 9–45 years was only approximately 2.24% during 2018–2020; the first-dose coverage increased to 10.15% by 2022 [10]. Provinces in eastern areas achieved higher coverage than those in western areas [11]. Among males, the vaccination rate remains persistently below 5% [12].

The situation started to change after China became the third country in the world with independent production and supply capability of HPV vaccines in 2019, and pilots of free HPV vaccination were initiated and continuously expanded thereafter, which leveraged the national implementation in 2025. In that year, China became the 155th country to provide free vaccination to all 13-year-old girls with two doses of the domestic bivalent HPV vaccine. Free catch-up vaccination is also offered to girls aged 14–18 [13]. This progression exemplifies a bottom-up policy evolution from local pilots to national implementation. In a country with the largest population and socioeconomic disparities, this unique approach served as a cornerstone for the national policy transformation. The policy change in China aligns with the Immunization Agenda 2030, which aims to increase domestic resource mobilization for immunization and ensure sustained government financing in developing countries [14]. Documentation of the process and summarization of what was learnt from China would offer valuable insights for NIP expansion in the other developing countries, particularly for those no longer eligible for external donor funding for immunization.

Given that the HPV vaccine was just introduced into the Chinese NIP, and local pilots have not yet been systematically reviewed, rigorously evaluated, and published, this review was performed primarily based on the information and data collected from the official websites of Chinese municipal, provincial, and central governments and their technical arms, in the areas of public health, disease prevention and control, maternal and child health, and health insurance. These government agencies publicly released official policy documents about the free HPV vaccination pilots, implementation plans, progress reports, experience sharing documents, and HPV vaccination coverage data. These materials helped to perform the narrative review of the initiation, evolution, and expansion of the local pilots. The official websites of relevant international and national organizations were also the key sources of global policy and progress of HPV vaccination, as well as vaccination coverage data, including the WHO, Catalan Institute of Oncology/International Agency for Research on Cancer Information Centre on HPV and Cancer (ICO/IARC HPV Information Centre), Innovation Lab for Vaccine Delivery Research, and Cancer Foundation of China. This information was mainly used in the background review and discussion, referring to the global progress of free HPV vaccination and vaccination coverage. The practices of free HPV vaccination in other countries were identified from searching published academic literature and generally highlighted the outstanding practices in these countries. A summary table of the main websites included in the analyses is presented in Table S1. In addition, the authors also integrated insights from key informants across central and local governments and institutions, leading academicians specialized in prevention and control of cervical cancer and promotion of HPV vaccination through semi-structured interviews, as well as findings from the field investigations and discussions in two provinces (Figure S1). These helped us to understand the driving force, the impacts, and the implications of the local pilots, as well as to analyze the enablers and barriers for the free HPV vaccination pilot and national implementation. Guangdong province pioneered the first provincial-level free HPV vaccination, and Shandong province significantly reduced the price of the domestic bivalent HPV vaccine through provincial pooled procurement. The HPV vaccines that are marketed in China, with their approved subtypes, are listed in Table S2. A list of key informants and semi-structured interview topics is provided in Supplementary Text S1. Data on disease burden and vaccination coverage were collected from formal published peer-reviewed literature and cited in the introduction to describe the baseline situation. The policy formulation process of free HPV vaccination in China aligns with Kingdon’s Multiple Streams Framework (MSF), which is a theoretical lens to enable a comprehensive examination of agenda-setting, policy entrepreneurship, and policy change processes across domains with a specific focus on the three core streams of problem, policy, and politics [15]. As critical policy changes materialize only when multiple streams converged within a policy window, which creates opportunities for agenda-setting and decision-making, we had the key facts and insights obtained from the literature review, the key informant interview, as well as the field investigations and discussions sorted out into three streams—problems, policies, and politics—which helped us to understand how the local pilots and subsequent expansion created a policy window for policy change at the national level.

## 2. Evolution of the Free HPV Vaccination Policy in China

**Figure 1:**
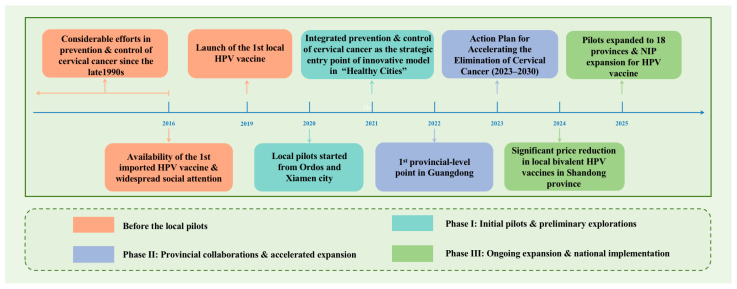
Milestones on the journey of free HPV vaccination policy in China.

Unlike traditional NIP expansion, which adopts a top-down decision-making strategy, China’s free HPV vaccination policy evolved with a distinct bottom-up strategy, originating from local pilots. The success of this strategy indicates that local pilots were instrumental to national policy-making. As highlighted in Figure 1, there were several key milestones achieved on the journey to the free HPV vaccination policy in China. First of all, the breakthrough was owed to the joint unremitting efforts of a group of dedicated clinical and public health experts in pushing an integrated strategy for the prevention and control of cervical cancer, i.e., prevention through social mobilization, health education, and HPV vaccination, and early detection through the expansion of screening and standardized treatment. Considerable education and advocacy efforts have been made since the late 1990s [16]. With the first imported HPV vaccine available in 2016, HPV vaccination gained widespread societal attention in China [17].

In the initial phase, Ordos was found to have the highest incidence of cervical cancer through the large-scale screening of 120,000 women during 2005–2010 [18]. Screening and treatment did not completely change the situation until the turning point occurred, when the first domestic HPV vaccine was launched in China in 2019. These milestones amplified the public awareness of HPV vaccination. The media coverage further elevated its visibility. Scientific evidence boosted the confidence of the health officials of Ordos to initiate the free HPV vaccination pilot through modeling the scenarios via which cervical cancer elimination can be achieved in China in the late 2050s, provided that the budget was increased for the HPV vaccination of girls aged 12 years and the screening of HPV infections was expanded [19,20].

The launch of the first domestic HPV vaccine in China directly stimulated the local pilots, which provided free HPV vaccination primarily to high-school girls aged 13 and above, through local government-led welfare projects with funding and institutional support. Some areas allowed insured individuals to use their personal medical savings accounts to cover HPV vaccination costs for themselves and their immediate family members (spouses, children, siblings, and grandchildren) [21]. Local public health insurance contributed to the free HPV vaccination as an alternative source of funding, although preventive vaccines are funded by a dedicated government budget in China [22].

One year later, the National Patriotic Health Campaign Committee and Healthy China Action Promotion Committee prioritized the integrated prevention and control of cervical cancer as the strategic entry point for an innovative “Healthy Cities” development model [16]. A batch of such “Healthy Cities” emulated the above pilots, and evolved as demonstrations for the other cities to follow successively.

Entering the subsequent phase, Guangdong became the first province to offer free HPV vaccination to all eligible girls within the entire jurisdiction in 2022. The key enablers of the pilot in Guangdong were strong political support from its senior provincial leadership and robust governmental financial capacity. Same as the other pilots, Guangdong designated free HPV vaccination as part of its provincial government-led welfare projects [23]. The significant welfare features of the pilots fostered the transcendence of the original scope of the “Healthy Cities” and propelled a rapid expansion across the country. In 2023, the central government explicitly expressed support for free HPV vaccination in well-prepared regions [24]. Even in resource-scarce areas, some local governments utilized limited public resources to implement this policy targeting the rural girls first [25,26].

The year 2024 marked another milestone characterized by substantial geographic expansion. The growing committed demand of domestic bivalent HPV vaccines enabled the achievement of economies of scale, which triggered a significant price reduction. Shandong, one of the most populous provinces in China, obtained the most significant price reduction in the domestic bivalent HPV vaccine through its provincial pooled procurement. The price fell to less than USD 4 per dose, falling within the acceptable threshold for NIP expansion, which brought a critical momentum for the introduction of HPV vaccines into the NIP. By the end of September 2025, when the decision regarding NIP expansion was made, the free HPV vaccination policy had already been implemented in 18 provinces (Figure S1) and many prefecture-level cities [16].

## 3. Problem, Policy, and Politics Streams of the Free HPV Vaccination Policy

**Figure 2:**
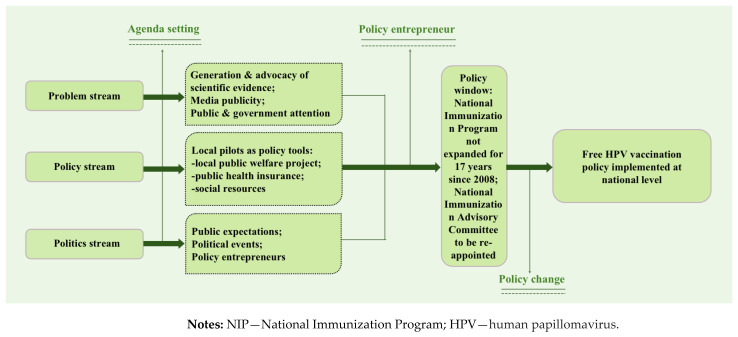
Problem, policy, and politics streams of the bottom-up policy evolution.

As shown in Figure 2, the problem stream that identified critical issues demanding policy attention for HPV vaccination had been pooled before the initiation of the first pilot in 2020. A group of leading clinical and public health experts served as pivotal actors in generating evidence and advocating for policy action. Evidence about the disease burden [6,7,27,28], socioeconomic impact of cervical cancer due to consistent HPV infections [29,30,31,32,33], and the irreplaceable role of HPV vaccination in moving towards the elimination of cervical cancer have been widely disseminated. This evidence attracted unprecedented attention from the public and health policy decision-makers.

The policy stream encompasses innovative solutions originated from the local pilots where local governments creatively incorporated the free HPV vaccination policy into the framework of their welfare projects, or those made possible through alternative funding mechanisms, including public health insurance and social resource contribution [21,34]. The free HPV vaccination policy evolved into a powerful policy instrument for many local policy-makers to demonstrate their achievements in enhancing public welfare and fostering public affinity.

Finally, the political stream includes the factors influencing policy decision-making, such as public opinions in mirroring the free HPV vaccination pilots, political events like collaborative effort in creating an innovative model for the construction of “Healthy Cities”, and the actions of different policy entrepreneurs like clinical and public health experts, as well as municipal, provincial, and central policy-makers. These players had made relentless efforts in turning the topic of free HPV vaccination into a social hotspot, informing decision-making, providing technical support for policy formulation, and driving its translation into practice.

The year of 2025 marked a pivotal convergence of the problem, policy, and politics streams, with a confluence of circumstances ripe for transformative change, and an alignment of factors that created an environment in which to enable change. The National Immunization Advisory Committee (NIAC) is scheduled for re-appointment for the first time since its establishment in 2017, and the NIP is to be expanded 17 years after its last update in 2008 [35]. The central decision-makers will seize this policy window to set the policy agenda for the nationwide implementation of the free HPV vaccination policy.

## 4. Profound Impacts of the Local Pilots

### 4.1. Rapid Increase in HPV Vaccination Rate

The extensive local pilots of free HPV vaccination have served as a powerful engine that drives a rapid and substantial increase in the HPV vaccination rate in China. As of September 2025, when the central government announced the NIP expansion for the HPV vaccine, over 60% of the targeted girls have been provided with free HPV vaccinations across the country [17].

### 4.2. Significant Price Reduction in Domestic Bivalent HPV Vaccines

The extensive local pilots of free HPV vaccination have served as a powerful engine that drives a rapid and substantial increase in the HPV vaccination rate in China. The expansion of local pilots played a pivotal role in shaping the market of HPV vaccines in China, and contributed to achieving economies of scale, which triggered a substantial price reduction. The price of less than USD 4 per dose for the domestic bivalent HPV vaccine is far below the lowest price of USD 10 per dose paid by the United Nations International Children’s Emergency Fund [36]. Existing evidence showed that when the price of the HPV vaccine went down to USD 5–10, NIP had an annual budget increase of 21.38% to 34.23% [37]. The price of USD 4 implies a less than 20% increase in the annual budget for introducing the HPV vaccine into the NIP.

### 4.3. Extensive Social Consensus Reached

The success of local pilots has fostered national consensus on the critical role of HPV vaccination in cervical cancer prevention and control, a principle now enshrined in the core public health knowledge repository across the country. Public health education and policy advocacy campaigns disseminated through diverse media platforms have been pivotal in driving this consensus. As an amplifier, educator, and legitimizer, the media has been playing a multifaceted and important role in promoting the free HPV vaccination policy, bridging the gaps between government policy, public awareness, and social acceptance. Recent surveys found that the awareness of cervical cancer prevention and control by women increased from less than 50% to over 90% in China [17].

## 5. Implications for NIP Expansion

With the increasing uncertainty of external donor funding for immunization, the issues surrounding new vaccine introduction and the sustainability of immunization coverage in developing countries have begun to receive widespread attention. Some countries that were formerly eligible for external donor funding are not adequately prepared to introduce new vaccines to the NIP in a timely manner [14,38]. The lessons learnt from China could provide the following insights for the other developing countries.

### 5.1. Adopting the Bottom-Up Strategy

Like most of the other countries worldwide, China has been adopting a conventional top-down strategy for decision-making regarding NIP expansion, which often requires strong central government advocacy. Local governments just need to follow the central decisions [39,40,41]. The decision-making process for introducing the HPV vaccine into the NIP adopted a unique bottom-up approach with a strong foundation of five-year local pilots [42]. As presented in Table 1, some local pilots adopted a similar bottom-up approach in sub-areas. The success and lessons learnt from such an innovative approach allowed the central government to carefully observe, think over, and recognize the necessity, urgency, feasibility, and challenges faced, which favor the approach of healthcare resource mobilization and allocation as well as the allowance of more flexibility to adapt to the evolving circumstances and incorporate perspectives of diversified stakeholders. This is particularly crucial for the decentralized settings in many developing countries with significant disparities in socioeconomic development and fiscal capacity, in which local governments play an increasingly important role in the financing and delivery of healthcare services.

### 5.2. Leverage of Comprehensive Platform Under the Framework of National Policy

The evolution of the free HPV vaccination policy in China is greatly attributed to the inter-departmental coordination in health, finance, education, and the protection of women’s rights. Mobilizing collaborative efforts in raising public awareness through cross-sector collaborations and strategic partnerships has been the key practical strategy of local pilots [43]. Positioning this policy within a broader policy framework broke the traditional leadership of the disease prevention and control program, leveraging the comprehensive platforms of Patriotic Health Campaign Initiative and Healthy China Action. Seeking policy breakthroughs requires leveraging and identifying synergies of relevant national policies.

### 5.3. Making Use of Effect of Scale and Peer Pressure

The transition of the free HPV vaccination policy reflects the demonstration effects. The outcomes and experiences of local pilots have been disseminated through regular experience-sharing activities organized by the National Cancer Center, and further amplified by media advocacy [44]. The effect of scale has been gradually achieved and further reinforced through snowballing expansions. The media has been playing a significant role in amplifying these effects. In the Chinese setting, representatives of the People’s Congress have the legal duty to propose public welfare motions. Free HPV vaccination has been one of the key focuses. Policy-makers outside the pilot areas are unwilling to fall behind in political performance evaluation, and therefore push forward the imitation of this policy. Public awareness also prompts the demands for analogous policies in people’s own jurisdictions. The effect of scale and peer pressure has been pushing more areas to follow.

### 5.4. High-Level Engagement and Cross-Departmental Collaboration

The effective rollout of local pilots is primarily contingent upon committed leadership and high-level cross-sectoral promotion. As an innovative model for the construction of a “Healthy City”, local pilots possess the inherent organizational and institutional strengths. These advantages include high-level government stewardship, multi-agency collaboration, and societal mobilization, which are instrumental for the endorsement and implementation of the government-led welfare projects. Policy progression has been further underpinned by support and guidance provided by the leading clinical and public health experts, who supplied essential data on disease burden, vaccination uptake, and cost–benefit analyses of various vaccination strategies [43,45], as the backup for decision-making. Compared to the rapid advancements at the local level, it is worth noting the inactive NIAC and delayed NIP expansion at the central level. The Technical Advisory Group (TAG) serves as an essential mechanism for guiding the introduction of new vaccines into the NIP and enhancing the scientific rigor and public credibility of vaccine policies [46]. Delayed re-appointment might negatively affect the functioning of NIAC and NIP expansion in China. Indonesia, another upper-middle-income country (UMIC), could provide more valuable insights on the positive role of TAG in introducing HPV and pneumococcal conjugate vaccines into the NIP [47].

### 5.5. Multiple Financing Mechanisms

Multiple financing mechanisms are essential for sustainable immunization, including either the cost-sharing of the government budget among different levels of government or alternative sources of funding besides the government budget. The NIP is financed dominantly by the central government in China, and the share of funding is affected by competing public health priorities. When NIP expansion encountered financial constraints, the central government encouraged well-prepared areas to pilot free HPV vaccination with local funds [48]. As presented in Table 1, the cost-sharing of the government budget among different levels of government tailored to local economic conditions has been adopted in all four local pilots. The cost-sharing of the government budget is particularly important for places with uneven socioeconomic development, as in many developing countries, where NIP financing is distributed among central, provincial, and district channels, and local governments have been asked to bear more of the cost, aiming to reduce the fiscal burden on the central government [38].

Given the growing uncertainties regarding external donor funding, many countries that were previously eligible for external financial support have had to self-finance the NIP. Although a government budget is the predominant financing mechanism of the NIP in most developing countries, an increasing number of them have been developing public health insurance systems, considering it as an alternative source of funding for immunization. As shown in Table 1, payment for HPV vaccination by public health insurance in Xiamen has been a supplement for the government-led welfare project to fund free HPV vaccination. Malaysia, another UMIC, also explored innovative financing mechanisms, including health insurance schemes and public–private partnerships, to secure long-term funding for vaccination programs [38].

## 6. Conclusions

The journey of bottom-up policy formulation and implementation exemplifies a quintessential model of policy making for NIP expansion in developing countries. The strategies include starting with local experimentation, expanding progressively, leveraging the relevant comprehensive platform under the framework of national policy, utilizing the effect of scale and peer pressure, high-level promotion, cross-departmental collaboration, and multiple financing mechanisms.

## Figures and Tables

**Table 1:** Features of four local pilots.

	Geographical Location	Rank of Economic Development	Start Time of the Pilot	Milestone Achievement	Bottom-up Strategy Within the Pilot Region	Budget Sharing	Public Health Insurance as Alternative Financing
**Ordos city**	North China	High	2020	One of 1st local pilots, demonstration for “Healthy City”	✓	✓	×
**Xiamen city**	East China	High	2020	One of 1st local pilots, demonstration for “Healthy City”	×	✓	✓
**Guangdong** **province**	South China	High	2022	1st local pilot within provincial jurisdiction	✓	✓	×
**Shandong** **province**	East China	High	2024	Significant price reduction of domestic bivalent vaccine through provincial pooled procurement	✓	✓	×

## Data Availability

No new data were created or analyzed in this study. Data sharing is not applicable to this article.

## Supplementary Materials

The following supporting information can be downloaded at: https://www.mdpi.com/article/10.3390/vaccines14060528/s1, Table S1. Summary table of the main websites included into the analyses; Table S2. HPV vaccines marketed in China by June 2026 with approved sub-types; Figure S1. Pilot areas of free HPV vaccination in China by September 2025; Supplementary Text S1. Semi-structured key informant interview topics.

## Abbreviations

HPV	human papillomavirus
NIP	National Immunization Program
WHO	World Health Organization
MSF	Multiple Streams Framework
NITC	National Immunization Advisory Committee
TAG	Technical Advisory Group
UMIC	upper-middle-income country
