# Supplementary material for: From Local Pilots to National Implementation: A Journey Towards Free HPV Vaccination in China
Source: Vaccines (Basel). 2026 Jun 15;14(6):528. doi: 10.3390/vaccines14060528 (PMC13307851; doi:10.3390/vaccines14060528)
Supplement: Supplementary file 1 [file vaccines-14-00528-s001.zip › vaccines-4345740-supplementary.pdf]

Supplementary file

From local pilots to national implementation:

a journey towards free HPV vaccination in China

Table S1. Summary table of the main websites included into the analyses.

	Web Address	Organization	Data provided by the website	Note
1	https://www.who.int/	World Health Organization	Global cervical cancer elimination strategy; HPV vaccination targets; country-level HPV vaccination coverage data;	The website of international organization
2	https://hpvcentre.net	ICO/IARC HPV Information Centre	China's HPV-related disease data	The website of international organization
3	https://www.ndcpa.gov.cn/	National Disease Control and Prevention Administration	Notice on the inclusion of HPV vaccine in China's National Immunization Program	The website of China's central government
4	https://www.nhc.gov.cn/	National Health Commission of the People's Republic of China	Process of introducing HPV vaccination into China's National Immunization Program	The website of China's central government
5	https://wjw.beijing.gov.cn/	Beijing Municipal Health Commission	One of PLADs piloted free HPV vaccination before national implementation	The website of China's local government
6	https://wsjk.tj.gov.cn/	Tianjin Municipal Health Commission	One of PLADs piloted free HPV vaccination before national implementation	The website of China's local government
7	https://wjw.jiangsu.gov.cn/	Jiangsu Commission of Health	One of PLADs piloted free HPV vaccination before national implementation	The website of China's local government
8	https://wjw.ah.gov.cn/	Health Commission of Anhui Province	One of PLADs piloted free HPV vaccination before national implementation	The website of China's local government
9	http://wsjkw.shandong.gov.cn/	Health Commission of Shandong Province	One of PLADs piloted free HPV vaccination before national implementation	The website of China's local government
10	https://wsjkw.zj.gov.cn/	Health Commission of Zhejiang Province	One of PLADs piloted free HPV vaccination before national implementation	The website of China's local government
11	https://hc.jiangxi.gov.cn/	Health Commission of Jiangxi Province	One of PLADs piloted free HPV vaccination before national implementation	The website of China's local government

12	https://wjw.fujian.gov.cn/	Health Commission of Fujian Province	One of PLADs piloted free HPV vaccination before national implementation	The website of China's local government
13	https://wst.hainan.gov.cn/swjw/index.html	Health Commission of Hainan Province	One of PLADs piloted free HPV vaccination before national implementation	The website of China's local government
14	http://ynswsjkw.yn.gov.cn/	Health Commission of Yunnan Province	One of PLADs piloted free HPV vaccination before national implementation	The website of China's local government
15	https://wsjkw.sc.gov.cn/	Health Commission of Sichuan Province	One of PLADs piloted free HPV vaccination before national implementation	The website of China's local government
16	http://wsjkw.cq.gov.cn/	Health Commission of Chongqing Province	One of PLADs piloted free HPV vaccination before national implementation	The website of China's local government
17	https://wjw.hubei.gov.cn/	Health Commission of Hubei Province	One of PLADs piloted free HPV vaccination before national implementation	The website of China's local government
18	https://wsjkw.nx.gov.cn/	Health Commission of Ningxia Hui Autonomous Region	One of PLADs piloted free HPV vaccination before national implementation	The website of China's local government
19	http://wsjk.gansu.gov.cn/	Health Commission of Gansu Province	One of PLADs piloted free HPV vaccination before national implementation	The website of China's local government
20	https://wjw.xizang.gov.cn/	Health Commission of Tibet Autonomous Region	One of PLADs piloted free HPV vaccination before national implementation	The website of China's local government
21	https://cdcp.gd.gov.cn/	Guangdong Provincial Center for Disease Control and Prevention	China's first provincial-level free HPV vaccination pilot; One of PLADs piloted free HPV vaccination before national implementation	The website of China's local government
22	http://www.gxzf.gov.cn/	Guangxi Zhuang Autonomous Region People's Government	Resource-scarce areas implementing free HPV vaccination pilots; One of PLADs piloted free HPV vaccination before national implementation	The website of China's local government
23	https://www.ordos.gov.cn/	Ordos Municipal People's Government	Process of the Ordos pilot	The website of China's local government
24	https://hfpc.xm.gov.cn/	Health Xiamen Action Promotion Committee	Process of the Xiamen pilot	The website of China's local government
25	https://www.nanjing.gov.cn/	Nanjing Municipal People's Government	Information on an alternative funding source for HPV vaccination	The website of China's local government

26	https://www.songpan.gov.cn/	Songpan County People's Government	Resource-scarce areas implementing free HPV vaccination pilots	The website of China's local government
27	https://www.yaan.gov.cn/	Ya'an Municipal People's Government	Resource-scarce areas implementing free HPV vaccination pilots	The website of China's local government
28	https://xxgk.jcgov.gov.cn/	Jincheng Municipal Health Commission	An example of pilot cities	The website of China's local government
29	Cancer Foundation of China	Cancer Foundation of China	Role of social organizations in the inclusion of HPV vaccination in China's National Immunization Program	The website of social organization
30	https://dce.cicams.ac.cn/	Chinese Academy of Medical Sciences	Role of clinical and public health experts in the inclusion of HPV vaccination in China's National Immunization Program	The website of academic institution
31	https://vaxlab.dukekunshan.edu.cn	The Innovation Lab for Vaccine Delivery of Kunshan Duke University	Information on an alternative funding source for HPV vaccination; information on Technical Advisory Group (TAG)	The website of academic institution
32	https://www.bjnews.com.cn/	The Beijing News	Process of the Ordos pilot	The website of social media

Notes: PLADs, provincial-level administrative divisions.

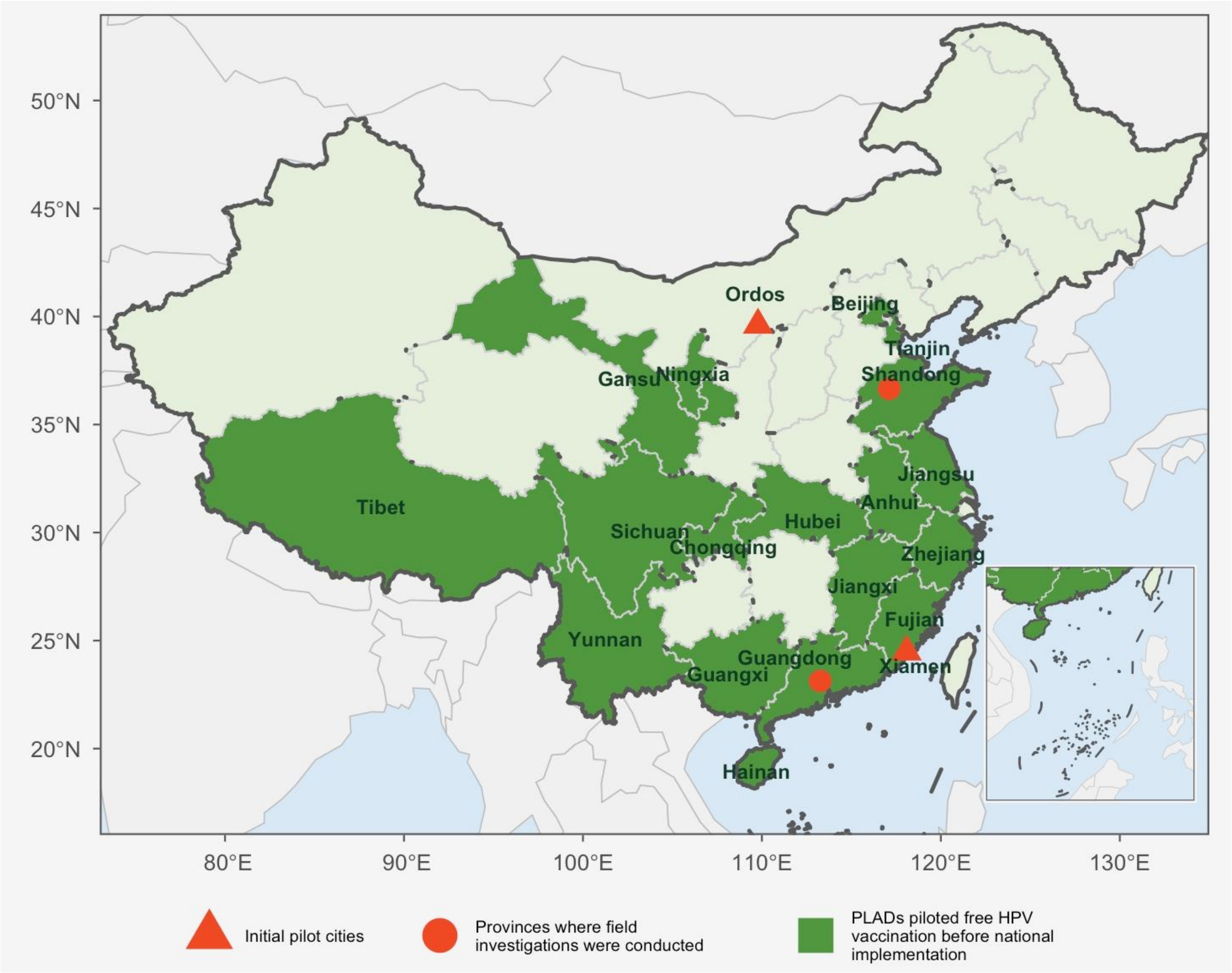

Figure S1. Pilot areas of free HPV vaccination in China by September 2025.

Notes: 18 provincial-level administrative divisions (PLADs): Guangdong, Fujian, Hainan, Jiangsu, Zhejiang, Jiangxi, Shandong, Hubei, Chongqing, Tibet, Guangxi, Sichuan, Ningxia, Gansu, Anhui, Beijing, Tianjin, and Yunnan.

Table S2. HPV vaccines marketed in China by June 2026 with approved sub-types.

	**Brand name**	**Manufacturer**	**Year of approval in China**	**Sub-types**
**Bivalent HPV vaccine (baculovirus expression system)**	Cervarix	GlaxoSmithKline, United Kingdom	2016	16/18
**Quadrivalent HPV vaccine (Saccharomyces cerevisiae)**	Gardasil	Merck & Co., Inc., United States	2017	6/11/16/18
**9-valent HPV vaccine (Saccharomyces cerevisiae)**	Gardasil9	Merck & Co., Inc., United States	2018	6/11/16/18/31/33/45/52/58
**Bivalent HPV vaccine (Escherichia coli)***	Cecolin	Xiamen Innovax Biotech Co., Ltd., China	2019	16/18
**Bivalent HPV vaccine (Pichia pastoris)**	Walrinvax	Yunnan Walvax Biotechnology Co., Ltd., China	2022	16/18
**9-valent HPV vaccine (Escherichia coli)**	Cecolin9	Xiamen Innovax Biotech Co., Ltd., China	2025	6/11/16/18/31/33/45/52/58
**Quadrivalent HPV vaccine (Hansenula polymorpha)**	Aiweijia	Chengdu Institute of Biological Products Co., Ltd.	2025	6/11/16/18

Supplementary text S1. Semi-structured key informant interview topics

1. **General background**

   HPV vaccination coverage;

   Local HPV vaccination policy description

2. **Initiation and plan**

   Rationale and driving factors, key policy objective;

   Challenges and solutions in policy formulation process;

   Public awareness and acceptance of HPV vaccination

3. **Implementation and management**

   Division of tasks and responsibilities of each department;

   Source of funding;

   Venue, procedure of vaccination;

   Selection and procurement of vaccines

   Training and performance assessment of health professional; Public education and advocacy;

   Challenges and solutions in implementation and management

4. **Impact and implication**

   Vaccination rate;

   Enablers and barriers to implement the free HPV vaccination policy;

   Sustainability and solutions

Participants, time and venue of the key informant interviews in provinces

Note: Guangdong province pioneered the first provincial-level free HPV vaccination, and Shandong province significantly reduced the price of domestic bivalent HPV vaccine through provincial pooled procurement.

1. **Face to face discussions in Guangdong province**

Time: Morning of 29 October 2024

Venue: Office of the Government of Guangdong Province

Participants: Social Security Section, Department of Finance, Guangdong Province

Maternal & Child Health Section, Department of Health, Guangdong Province

Immunization Institute, Center for Disease Control, Guangdong Province

Development Research Center, State Council

School of Health Policy and Management, Peking Union Medical College

Recorder: Zhitao Wang, School of Health Policy and Management, Peking Union Medical College

Key contents:

- Background of initiation of the pilot of free HPV vaccination in Guangdong province;
- Driving force of the pilot project;
- Details of the pilot project in terms of leading organization and coordination, financing and monitoring.

Time: Afternoon of 29 October 2024

Venue: Health Service Center of Shibei community, Tianhe district, Guangzhou city

Yutao School, Tianhe district, Guangzhou city

Participants: Social Development and Research Center, Guangdong province

Maternal & Child Health Section, Department of Health, Guangzhou city

Immunization Institute, Center for Disease Control, Guangzhou city

Maternal & Child Hospital, Guangzhou Medical University

Social Security Section, Department of Finance, Guangzhou city

Health Promotion Center, Primary and High School of Guangzhou city

Family Development and Maternal & Child Health, Department of Health, Tianhe district

Health Service Center of Shibei community, Tianhe district

Yutao School of Tianhe district

Development Research Center, State Council

School of Health Policy and Management, Peking Union Medical College

Recorder: Zhitao Wang, School of Health Policy and Management, Peking Union Medical College

Key contents:

- Details of the implementation of the pilot project;
- Status, achievements and problems met;
- Enablers and barriers.

2. Face to face discussions in Shandong province

Time: Morning of 12 December 2024

Venue: Office of the Government of Shandong province

Health Service Center of Shunyu Road, Jinan city

Jinan Middle School

Participants: Department of Health, Shandong province

Center for Disease Control, Shandong province

Department of Education, Shandong province

Department of Finance, Shandong province

Women Federation, Shandong province

Government Procurement Center of Shandong province

Development Research Center, State Council

School of Health Policy and Management, Peking Union Medical College

Recorder: Yihan Fu, School of Health Policy and Management, Peking Union Medical College

Key contents:

- Background of initiation of the pilot of free HPV vaccination in Guangdong province;
- Details of the pilot project in terms of leading organization and coordination, financing and monitoring;
- Details of the implementation of the pilot project;
- Provincial pooled procurement of HPV vaccines;
- Status, achievements and problems met;
- Enablers and barriers

Time: Afternoon of 12 December 2024

Venue: Office of the Government of Jinan city

Participants: Department of Health, Jinan city

Center for Disease Control, Jinan city

Department of Education, Jinan city

Department of Finance, Jinan city

Women Federation, Jinan city

Procurement agency

Guangchuan community, Decheng, Jinan city

Development Research Center, State Council

School of Health Policy and Management, Peking Union Medical College

Recorder: Yihan Fu, School of Health Policy and Management, Peking Union Medical College

Key contents:

- Background of initiation of the pilot of free HPV vaccination in Guangdong province;
- Details of the pilot project in terms of leading organization and coordination, financing and monitoring;
- Details of the implementation of the pilot project;
- Status, achievements and problems met;
- Discussion of HPV vaccination hesitancy

3. Other key informants

Officers from the following national government agencies and their technical arms:

National People's Congress

National Health Commission

Immunization Center of China Center for Disease Control and Prevention

Maternal and Child Health Center of China Center for Disease Control and Prevention

National Cancer Center

National Healthcare Security Administration

Key contents:

- The Healthy City initiative and the Health China 2030 national plan;
- How and why the free HPV vaccination policy was adopted by the above two important initiatives and linked closely with the maternal and child health program and the cancer prevention/control program;
- How and why did the Ordos pilot initiated the pilot of free HPV vaccination first in China;
- How did central government support the expansion of free HPV vaccination pilots;
- What is the process of inclusion of new WHO recommended vaccines into the national immunization program;
- Any feasibility analysis had been performed to include HPV vaccine;
- Local HPV vaccination coverage monitoring;
- Should the national health insurance fund be the key source of funding for national free HPV vaccination?
- National pooled procurement of HPV vaccines;
- Key barriers for implementing the free HPV vaccination policy at national level in China;
- Implications of national free HPV vaccination policy to include other WHO recommended vaccines into the national immunization program.
